# Failure to experimentally infect 10 days-old piglets with a cell culture-propagated infectious stock of a classical genotype 1a porcine epidemic diarrhea virus

**DOI:** 10.3389/fvets.2023.1279162

**Published:** 2023-11-16

**Authors:** Priscilla F. Gerber, Dianjun Cao, Chao-Ting Xiao, Qi Chen, Kelly Lager, Berend Jan Bosch, Xiang-Jin Meng, Patrick G. Halbur, Tanja Opriessnig

**Affiliations:** ^1^Department of Infectious Diseases and Public Health, City University of Hong Kong, Kowloon, Hong Kong SAR, China; ^2^College of Veterinary Medicine, Long Island University, New York, NY, United States; ^3^Hunan Provincial Key Laboratory of Medical Virology, Institute of Pathogen Biology and Immunology, College of Biology, Hunan University, Changsha, China; ^4^Department of Veterinary Diagnostic and Production Animal Medicine, College of Veterinary Medicine, Iowa State University, Ames, IA, United States; ^5^National Animal Disease Center, United States Department of Agriculture-Agricultural Research Services, Ames, IA, United States; ^6^Virology Section, Infectious Diseases and Immunology Division, Department of Biomolecular Health Sciences, Faculty of Veterinary Medicine, Utrecht University, Utrecht, Netherlands; ^7^Department of Biomedical Sciences and Pathobiology, College of Veterinary Medicine, Virginia Polytechnic Institute and State University, Blacksburg, VA, United States; ^8^Vaccines and Diagnostics Department, Moredun Research Institute, Penicuik, United Kingdom

**Keywords:** porcine epidemic diarrhea virus, PEDV, CV777 strain, virus attenuation, pig model

## Abstract

**Introduction:**

Porcine epidemic diarrhea virus (PEDV) causes enteric disease in pigs of all ages. PEDV can be grouped into G1 (classical strains) and G2 (variant strains) based on sequence differences in the spike gene. Although several pathogenesis studies using contemporary strains of PEDV have been conducted to date, there is limited information on the pathogenesis of historical PEDV strains in contemporary pigs. This study aimed to investigate the clinical disease course of 10 days-old pigs infected with a classical European G1a PEDV strain from the 1980s which was last passaged in pigs in 1994.

**Methods:**

Sequencing results confirmed that the virus inoculum was a PEDV strain closely related to the prototype CV777 strain. The PEDV stock was serially passaged three times in Vero cells, and the P3 infectious virus stock was used to inoculate the pigs. A total of 40 pigs were inoculated using the oral route.

**Results:**

Pigs showed no enteric disease signs, and PEDV shedding was not detected for 44 days post-inoculation (dpi). At necropsy at 3 (5 pigs) or 7 dpi (5 pigs), no lesions were observed in intestinal sections, which were negative for PEDV antigen by immunohistochemistry. In addition, no IgG or IgA PEDV-specific antibodies in serum or fecal samples for 35 dpi further indicates a lack of infection. Titration of the leftover thawed and refrozen PEDV virus stock inoculum showed that the virus stock retained its infectivity in Vero cell culture and the porcine small intestine enterocytes cell line IPEC-J2.

**Discussion:**

The reasons for the loss of infectivity in pigs are unknown. In conclusion, we showed that a classical G1a PEDV strain successfully propagated in cell cultures could not orally infect 40 piglets.

## 1 Introduction

Porcine epidemic diarrhea virus (PEDV), a member of the genus *Alphacoronavirus* in the family *Coronaviridae*, is highly contagious and causes an enteric disease characterized by an acute onset of vomiting and diarrhea in pigs of all ages (1). Coronaviruses consist of a large (~30 kb) positive sense single-strand RNA, with the first two-thirds of the genome encoding for nonstructural proteins involved in virus replication and host interactions (e.g., immune evasion), while the remaining third encodes for four major structural proteins: spike (S), envelope (E), membrane (M), and nucleocapsid (N) (2). Based on amino acid differences in the N-terminal domain of the S protein, PEDV is divided into two genogroups, G1 and G2, that can be further divided into G1a (CV777 and other classical strains), G1b (cell-culture adapted and other recombinant strains), G2a (US-like pandemic PEDV strains), G2b (Asian strains) and G2c [S-INDEL (variant S protein containing insertions and deletions) strains from the US, Europe, and China] (3, 4).

In the late 1970s, a diarrheic syndrome characterized by acute watery diarrhea in pigs of all ages, porcine epidemic diarrhea (PED), was described in Europe, causing outbreaks with mortality rates in suckling piglets ranging from 0%–100% (average 50%) with the severity of disease depending upon litter and farm (5). One of the 1970s PEDV isolates, CV777, became the PEDV prototype strain and was used for pathogenesis studies in colostrum-deprived pigs (6). PEDV spread to Asia in the 1980s, and since 2010 it has caused severe epidemics in many Asian countries, mainly associated with PEDV G2 strains (3, 4, 7).

The G1b was first reported in China in 2011, spread through Asia, and was attributed to 2015–2016 outbreaks in grow-finish farms in Europe (3). PEDV G2b and G1b isolates similar to PEDV strains circulating in China in 2012 were introduced to the Americas, the Caribbean, and Ukraine in 2013 and 2014 (3, 8). Several pathogenesis studies have been performed using G1b and G2b strains (9–14), indicating that G1b strains are less pathogenic than G2b strains, although clinical signs vary considerably. However, there is limited information on the pathogenesis of classical PEDV strains in contemporary pigs (15). This study aimed to investigate the clinical disease course in 10 days-old pigs experimentally inoculated with a classical CV777-like G1a PEDV strain.

## 2 Methods

### 2.1 Virus origin

A G1a PEDV strain designated as CV777 was originally isolated in Belgium in the 1980s and was obtained for this study from the University of Utrecht, the Netherlands (16). In 1994, this isolate was propagated for the last time in cesarean-derived, colostrum-deprived piglets, the pigs showed diarrhea following infection and intestinal perfusate was collected and stored at −80°C.

### 2.2 *In vitro* virus propagation and titration

All laboratory work was approved by the Iowa State University Biosafety Committee (Approval number: 14-I-0018-A). The PEDV viral stock was passaged three times (CV777 P3) in Vero cells as described previously (17), and a total volume of 0.6 L of infectious virus inoculum stock was produced. Minimum essential medium (MEM) supplemented with 10% fetal bovine serum, 2 mM L-glutamine, 0.05 mg/mL gentamicin, 10 unit/mL penicillin, 10 μg/mL streptomycin, and 0.25 μg/mL amphotericin, tryptose phosphate broth (0.3%), yeast extract (0.02%), and trypsin 250 (5 μg/mL) was used for virus propagation (17). The infectious virus stock was titrated by both the plaque-forming unit (PFU) method as having an infectious titer of 5 × 10^4^ PFU/mL and by a fluorescence forming unit (FFU) method as having an infectious titer of 3.4 × 10^4^ FFU/mL (17).

### 2.3 Sequencing and genomic analysis

To amplify the genomic sequence of the CV777 P3 virus stock of PEDV, oligonucleotide primers were designed (Supplementary Tables S1, S2) and synthesized based on the conserved regions among the sequences of prototype CV777 (AF353511) and recent US PEDV isolates. Fragments covering most of PEDV genome for the CV777 P3 stock were amplified by RT-PCR using these primers with PfuUltra II high-fidelity DNA polymerase (Agilent, Santa Clara, CA). The RT-PCR products were purified from the agarose gel with Zymoclean™ Gel DNA Recovery Kit (Zymo Research Corp, CA) and sequenced directly by the Sanger method. Sequence contigs were assembled and analyzed using the Lasergene package (DNAStar, Inc., Madison, WI). The phylogenetic tree for the S protein was constructed by using the maximum-likelihood method with bootstrap tests of 1,000 replicates using DNAStar.

### 2.4 *In vitro* infection of swine small intestinal cells

An immortalized swine jejunum epithelial cell line IPEC-J2 (ACC 701, DSMZ, Braunschweig, Germany) was used as *in vitro* infection model. Fifty percent coverage of IPEC-J2 (cell passage 33) cell monolayer on the bottom wall of 24-well plates (Corning, Corning, New York, United States) was inoculated with 1 × 10^4^ FFU of PEDV CV777 P3 diluted in 1 mL PEDV propagation media as previously described (17), and section 2.2. After 48 h incubation, the appearance of cytopathic effect (CPE) was examined, and the cells were subsequently fixed with 80% acetone and stained with PEDV-specific monoclonal antibody SD6-29 conjugated to FITC (Medgene, Brookings, South Dakota) in an immunofluorescent assay (IFA) (18).

### 2.5 *In vivo* infection in 10 days-old pigs

The experimental protocol was approved by the Iowa State University Institutional Animal Care and Use Committee (Approval number 2-14-7804-S) and by the National Animal Disease Center, USDA-ARS Institutional Animal Care and Use Committee. Forty 10 days-old crossbred piglets negative for PEDV by ELISA (anti-PEDV IgA and IgG) and PCR bred at the National Animal Disease Center, USDA-ARS in Ames, Iowa were inoculated with the PEDV CV777 P3 infectious virus stock as part of another study (19). Each pig received 10 mL of the inoculum stock at PFU dose of 5 × 10^4^ per ml via the oral route by slowly dripping the inoculum into the oral cavity of the pig. After inoculation, approximately 10 mL of the remaining virus inoculum was frozen and stored at −80°C. The non-infected group (*n* = 40, no PEDV exposure) and the group infected with PEDV G2b strain US/Colorado/2013 at 10 days of age group (*n* = 43) described in the original study (19) served as negative and positive controls, respectively, to the current study.

### 2.6 Sample collection and storage

Blood samples were collected in serum separator tubes (Fisher Scientific, Pittsburgh, Pennsylvania, United States) at day post-PEDV inoculation (dpi) 0, 7, 14, 24, and 35 centrifuged at 3000 × g for 10 min at 4°C. Fecal samples were collected at dpi 35 and tested for anti-PEDV IgA antibodies. Fecal swabs were collected using polyester swabs daily from dpi 0 through 33 and dpi 44 and stored in 5 mL plastic tubes containing 1 mL of sterile saline solution. All samples were stored at −80°C until further testing.

### 2.7 Enzyme-linked immunosorbent assay

Serum samples tested for anti-PEDV IgA and IgG antibodies by an *in-house* S1-based indirect enzyme-linked immunosorbent assay (ELISA) (20, 21) and by an *in-house* G2b whole cell-based ELISA offered at Iowa State University Veterinary Diagnostic Laboratory (ISU-VDL) (18). Fecal samples were tested for anti-PEDV IgA antibodies by an *in-house* S1-based indirect ELISA (21).

### 2.8 RNA extraction and PCR assays

RNA was extracted from fecal swab suspensions using the MagMax Pathogen RNA/DNA Kit (Applied Biosystems, Life Technologies, Carlsbad, California, United States) and an automated DNA/RNA extraction system (Thermo Scientific Kingfisher Flex, Thermo Fisher Scientific, Pittsburgh, Pennsylvania, United States) according to the instructions of the manufacturer. Extracts were tested for PEDV RNA by an N gene-based quantitative reverse transcriptase (RT)-PCR offered at ISU-VDL and by an N-gene PEDV G1 and G2 differential real-time RT-PCR using the following pair of detection primers (PED-NDF: 5′-CGATGATCTGGTGGCTGCTGT-3′ and PED-NDR: 5′-GGGATGTCTTTGAGGTCACGTTC-3′) and TaqMan probes (PEDV-G1prob(CV777) CAL Fluor Orange 560-5′-TAAGCAGGAAAAGTCTGACAACAGCGGC-3′-BHQ and PEDV-G2prob FAM-5′-CAAACAGGAAAGGTCTGACAGCAGCGG-3′-BHQ).

### 2.9 Clinical signs

A daily diarrhea score on each pig was obtained by observing the pigs once every morning from dpi 0 to 8 and on dpi 10, 12, 14, 17, 18, 21, 24, and 35. Fecal composition was scored ranging from 0 to 3 (0 = normal feces, 1 = moist feces, 2 = pasty feces, 3 = watery feces).

### 2.10 Necropsy and sample collection

Five pigs were euthanized at dpi 3, 7 and 14 and the remaining pigs were euthanized at dpi 35 by sodium pentobarbital overdose (100 mg/kg of Fatal-plus, Vortech Pharmaceuticals, LTD, Dearborn, Michigan, United States). Death was confirmed by assuring cessation of respiratory and cardiovascular movements by observation before the pigs were necropsied. Eight sections of small intestines and three sections of large intestines were collected at necropsy and fixed in 10% neutral-buffered formalin and routinely processed for histological examination.

### 2.11 Microscopic lesions and immunohistochemistry

Microscopic lesions were evaluated by a veterinary pathologist blinded to treatment status (PGH) and evaluated for the presence of inflammation, villus atrophy, and necrosis. PEDV-specific antigen was detected by an IHC on selected formalin-fixed and paraffin-embedded sections of intestinal sections using monoclonal antibody specific for PEDV (BioNote, Hwaseong-si, Gyeonggi-do, Republic of Korea) as described (22, 23).

## 3 Results

### 3.1 The G1a PEDV virus inoculum used in this study is a classical strain related to CV777

**Figure 1:**
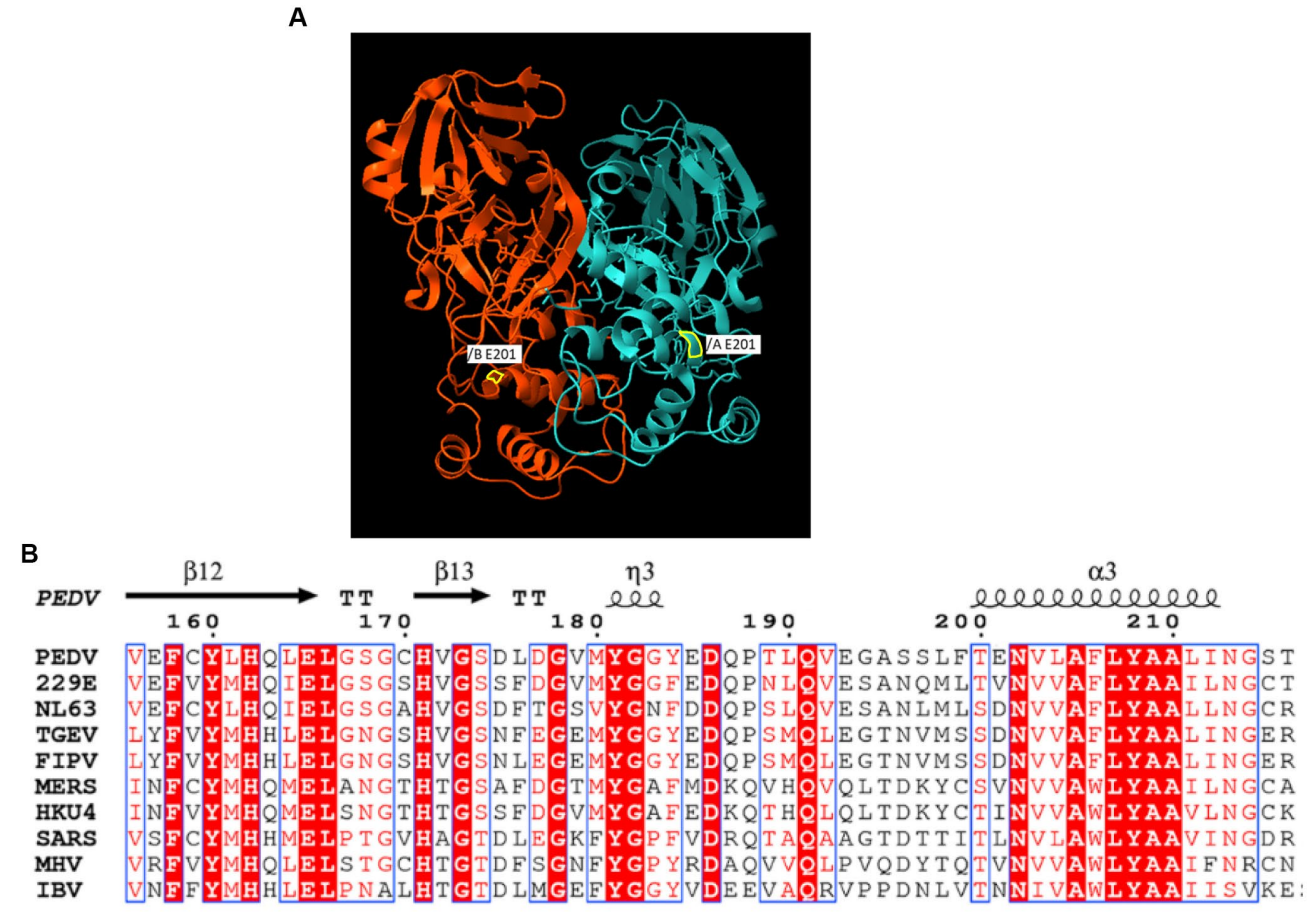
**(A)** The X-ray crystal structure of PEDV-3C-like protease structure (PDB entry 5HYO). A yellow border line indicates the location of E/D mutation. **(B)** Alignment of E/D mutation adjacent region of Coronaviruses 3C-like protease.

The sequence obtained for the near full-length genome of PEDV (GenBank No. OR348434) indicates that the G1a PEDV passage 3 virus is closely related to the prototype CV777 [GenBank No. AF353511, 99.9% nucleotide (nt) identity], and to a lesser extent to Asian G1a PEDV isolates such as CHM2013 (KM887144.1, China, 2013, 99.4% nt identity) and AVCT12 (LC053455.1, Thailand, 2015, 99.3% nt identity). When using the classical CV777 strain sequence as the reference, deletions in the 5′ noncoding region of the genome were identified, including a deletion at nt position 72 and a TCCT deletion at nt position 82–85, which is similar to G1a strain CHM2013 and strain AVCT12. There were two viral populations at the 9890 nt of the ORF1a in the CV777 P3 stock. The majority of the sequence population had a G to C mutation, which results in Glu (E) to Asp (D) amino acid mutation in the 3C-like protease. A GenBank BLAST search of CV777 P3 3C-like protease amino acid showed that the E/D mutation in the CV777 P3 virus is unique among known PEDV genomes. It is located within a helical chain rather than at the catalytic site. Figure 1 shows its position on the 3D structure of PEDV 3C-like protease, as well as an alignment of cDNA snippets from relevant viruses adapted from previous studies (24, 25). In addition, the ORF1b also had a deletion at 13077–13078.

**Figure 2:**
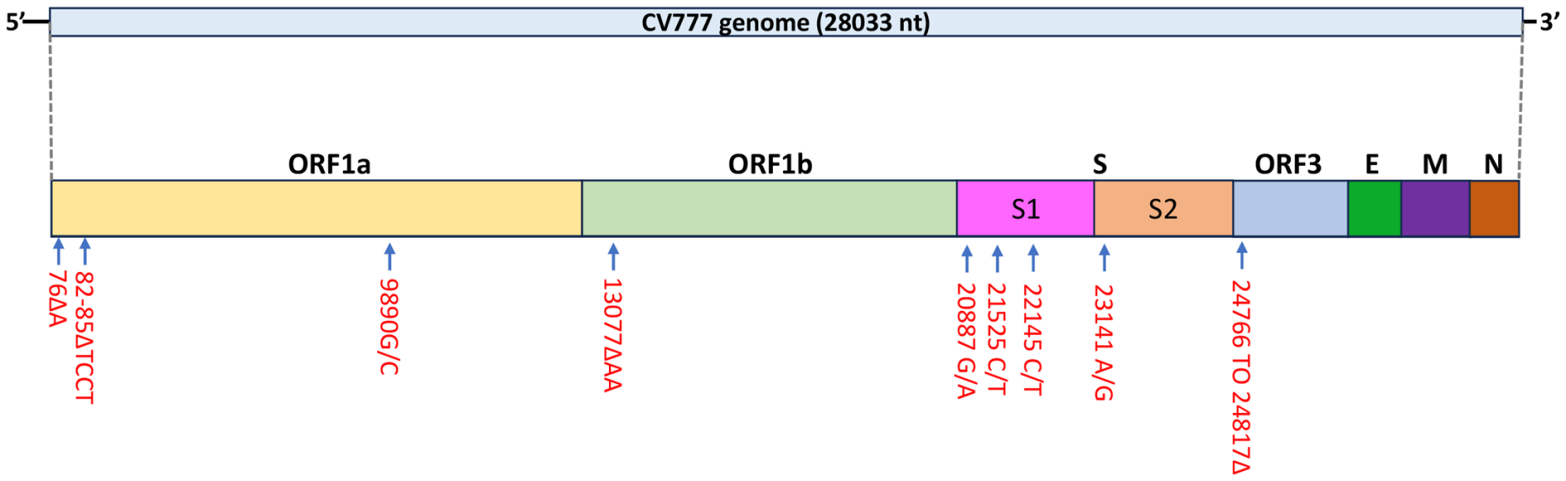
Schematic diagram of mutations identified in the PEDV CV777 P3 genome.

A total of four mutations were found in the S gene: 20887 G/A, 21525 C/T, 22145 C/T, and 23141 A/G and all mutations resulted in amino acid changes except for the mutation at nt position 21,525 (Figure 2).

**Figure 3:**
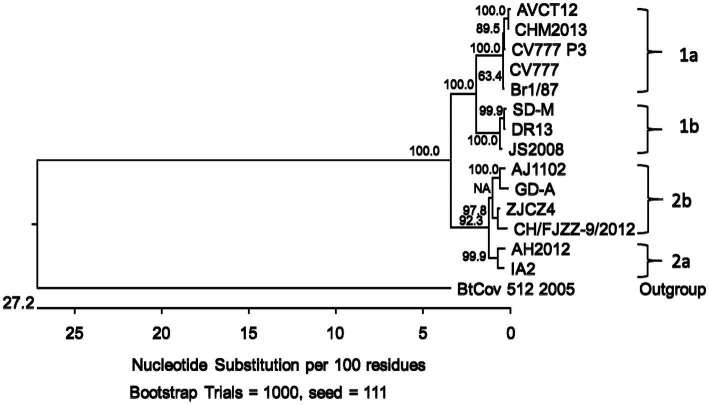
Phylogenetic tree based on the spike gene (S) translated amino acid sequences of porcine epidemic diarrhea virus strains from genogroups 1a, 1b, 2a and 2b. Bootstrap values are represented at key nodes.

Similar to the CHM2013 and AVCT12 strains, there was a 52 nt deletion between nt positions 24766 and 24817, which resulted in the truncation of the S protein by 7 amino acids and the deletion of the start codon of ORF3. According to the sequence, the deletion causes ORF3 to be truncated by 70 amino acids, leading to the production of peptides consisting of 154 amino acids. A C/T mutation was also found at nt position 25523. A phylogenetic tree based on the S protein amino acid sequences is shown in Figure 3.

### 3.2 G1a PEDV strain infected swine small intestinal epithelial cells *in vitro*

**Figure 4:**
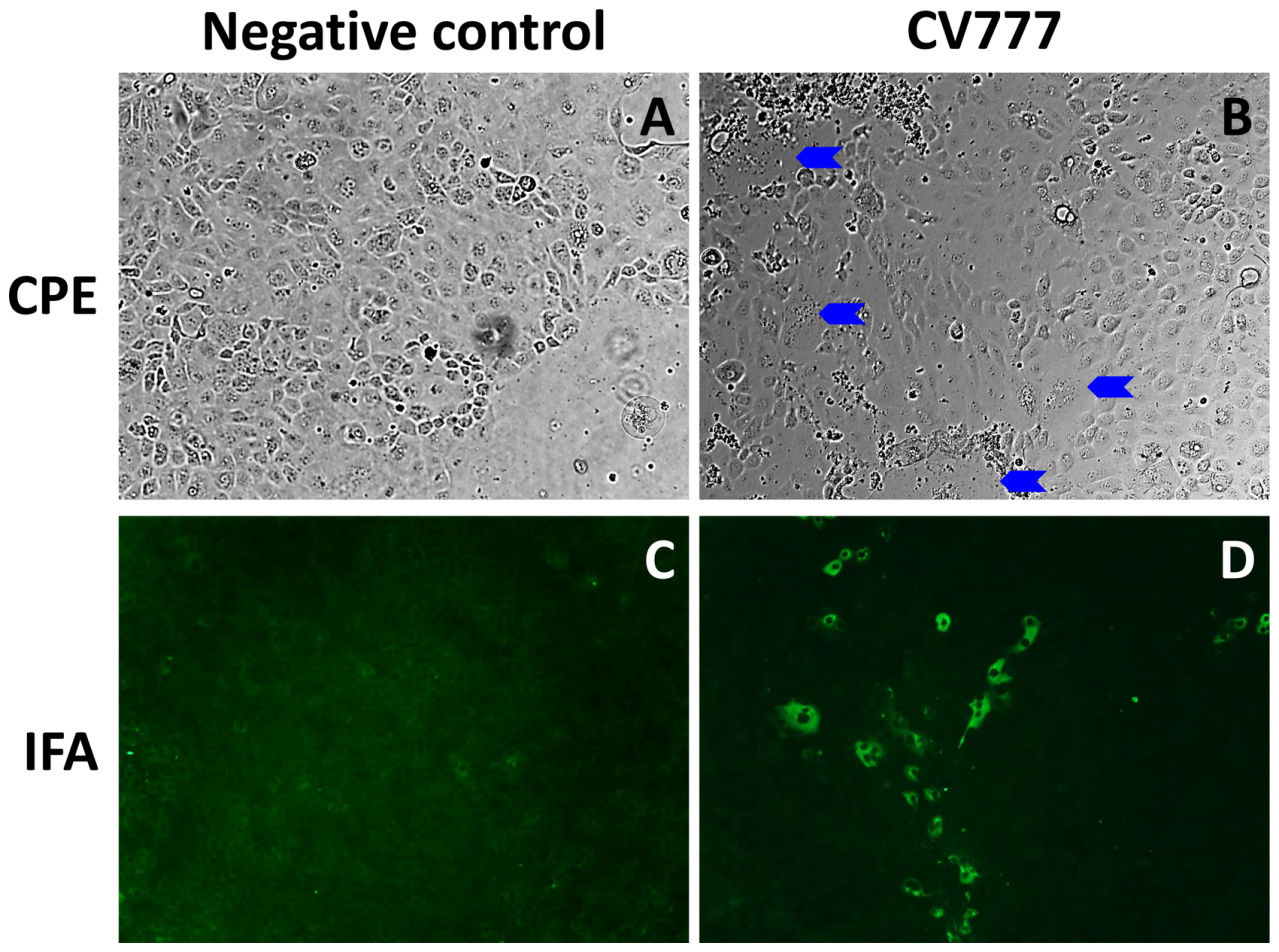
Cytopathic effect (CPE) and immunofluorescence staining (IFA) staining in IPEC-J2 cells at 48 h post inoculation. Mock-inoculated IPEC-J2 cells had no CPE **(A)** or IFA staining **(C)** observed. PEDV CV777 infected IPEC-J2 cell presented CPE (**B**, blue arrows: syncytial bodies) and IFA staining **(D)** (×40 magnification).

The PEDV CV777 P3 was infectious in Vero cells and the swine small intestinal epithelial cells IPEC-J2. After 48 h of inoculation, typical PEDV-induced CPE, multifocal cell syncytia presented as coalescing multinucleated cells with enlarged cytoplasm, were observed in PEDV CV777-inoculated IPEC-J2 cell monolayer (Figure 4). Additionally, PEDV viral proteins located in cytoplasm of infected IPEC-J2 cell were labeled and visualized with PEDV specific monoclonal antibodies conjugated to FITC (Figure 4).

### 3.3 Failure to experimentally infect pigs with the G1a PEDV strain

After inoculation, pigs were monitored daily for clinical signs and no diarrhea or other sign of PEDV infection was present in the inoculated piglets in accordance with the absence of PEDV RNA detection in fecal swabs for 35 days after inoculation. No lesions compatible with enteric disease were observed at necropsy or by microscopic examination on dpi 3 or 7. In addition, PEDV-specific antigen was not detected in the intestinal sections. No IgG or IgA PEDV specific antibodies were detected by dpi 35 in serum samples and no IgA PEDV specific antibodies were detected by dpi 35 in feces. Together, these results indicate that the piglets were not infected with the PEDV CV777 P3 related strain.

**Figure 5:**
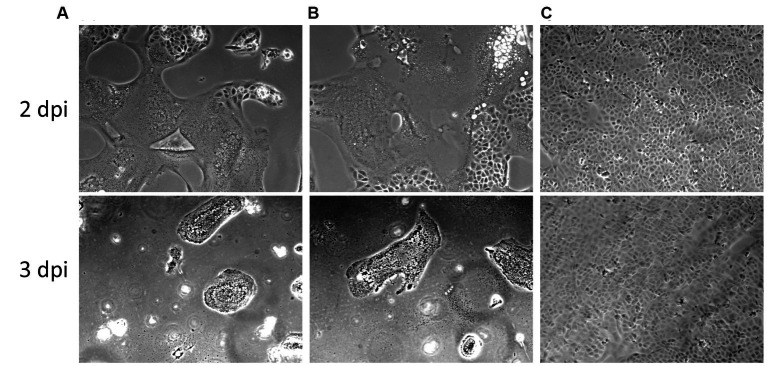
Cytopathic effects of the PEDV CV777 P3 used in this study in Vero cells at days post inoculation (dpi) 2 and 3. **(A)** Original viral stock. **(B)** Leftover inoculum previously used for inoculating 40 10 days-old pigs. **(C)** Mock infected cells.

To rule out the possibility of a potential loss of infectivity of the viral stock during storage, thawing and handling, we tested and re-titrated the leftover thawed and refrozen virus stock inoculum in Vero cells in parallel to the frozen virus inoculum stock. The thawed leftover inoculum still retained its infectivity in Vero cells at similar infectious titers compared to the unfrozen viral stock (Figure 5).

## 4 Discussion

In this study conducted in 2014, we found that a European PEDV isolate initially isolated from Belgium pigs in the 1970s (16), subsequently supplied to research laboratories for *in vitro* work failed to infect any of 40 10 days-old piglets experimentally-inoculated with a newly-prepared infectious stock of the virus. Titration of the PEDV virus stock that was used to inoculate the piglets and had been frozen back demonstrated that the virus stock retained its *in vitro* infectivity when titrated in Vero cells. Age-resistance to disease induced by PEDV infection has been reported and neonatal (1–9 days old) piglets often display more severe clinical signs than weaned (3–4 weeks old) pigs (10, 18). The piglets (10 days-old crossbred pigs) used herein should be highly susceptible to PEDV infection. In addition, contemporary piglets from the same source and housed in the same facilities were successfully infected with the G2b Colorado PEDV strain when a similar dose and infectious route were used in a previous study (19). The reason for the lack of the CV777-related PEDV infectivity to pigs is unknown, and unexpected. The G1a strain Br1/87 isolated in 1987 in Great Britain, closely related to the prototype CV777 (26), was successfully used to orally infect five 5 weeks-old conventional pigs in 2014 (15). Pigs experimentally infected with the Br1/87 strain had mild to moderate diarrhea and low levels of intermittent viral shedding in fecal swabs for 1–3 weeks (15). In addition, cell culture-attenuated as well as wild-type CV777 strain at a similar dose (2.55 × 10^5^ FFU/pig) used herein have been used successfully to infect conventional 11 days-old piglets in early 2000s (27).

Studies using cesarean-delivered colostrum-deprived (CDCD) or conventional piglets, performed in the late 1970s and early 1980s using classical G1a strains (6, 28) and a study using a 1990s Asian PEDV strain KPEDV-9 (29) reported similar clinical results to what has been reported in recent years for contemporary PEDV G1b and G2 strains (9, 10). In 2–3 days old CDCD piglets experimentally infected with PEDV strain CV777 in 1980s, viral particles were seen in the jejunum starting 18 h post infection (hpi) and clinical signs characterized by profuse watery diarrhea started 24–36 hpi (6, 28). Two hours after onset of clinical signs (approximately 24 hpi), exfoliation of enterocytes could be seen leading to severe villous atrophy while crypt epithelium was not affected (28). Pigs that did not die from dehydration 2–4 days after onset of diarrhea recovered after a week (6).

Although the CV777 P3 virus is closely related to the G1a PEDV prototype stain CV777, the mutations observed in the spike gene and 5′ noncoding region are similar to those observed in contemporary Asian strains CHM2013 and AVCT12, which is not surprising as modified live-attenuated vaccines similar to CV777 and Br1/87 have been used in Asia since the 1990s and these strains are still circulating in that region (3). It is possible that mutations have been acquired during the sequential *in vivo* passages that the original CV777 strain was subjected to throughout the years. However, it remains unknown why 10 days-old piglets were resistant to the CV777-like strain after being passaged only for three passages *in vitro*, especially since the CV777 P3 virus that was used in the pig study is fully infectious in Vero cells and in IPEC-J2 small intestinal epithelial cells. A genetically engineered PEDV with a deleted ORF3, similar to the deleted ORF3 pattern found in the present study, has been shown to successfully infect gnotobiotic piglets producing lethal disease outcomes and to infect Vero cells (30), indicating that the ORF3 is not essential for *in vivo* or *in vitro* replication. In addition, it has been shown that the loss of the N-domain portion of the S protein correlates with a loss of enteric tropism for some coronaviruses (31). For example, a naturally occurring variant of the transmissible gastroenteritis virus (TGEV), the porcine respiratory coronavirus (PRCV) lost its enteric tropism and mainly replicates in the respiratory tract after the loss of the N-domain portion of the S protein (32). The loss of enteric tropism for TGEV was specifically associated with the loss of the sialic binding activity that resides in this domain (31).

Interestingly, two populations of viruses could be found in the CV777 P3 virus stock, and the majority presented a G to C mutation at position 9890, which resulted in a mutation in the 3C-like proteinase gene leading to a truncated protein. This mutation is similar to the mutation described in the feline infectious peritonitis virus (FIPV), that replicates systemically in cats and has evolved as a deletion of the S-protein N-domain portion of the feline enteric coronavirus (FECV) which replicates in enterocytes (33). It has been shown that a complete 3C-like proteinase is required for the replication of FECV in the intestinal epithelium. Although the effect of this mutation on the infectivity of PEDV in pigs is unknown, it may have affected the tropism of the virus and contributed to the failure of oral infection in pigs. Therefore, it would be interesting to further determine the function of E/D mutation in the 3C-like proteinase of the CV777 P3 virus stock and if it affects virus infection in pigs.

## 5 Conclusion

We demonstrated that a classical G1a PEDV strain, although infectious in Vero and IPEC-J2 cells *in vitro*, was unable to infect 40 piglets by oral route of inoculation. The mechanism for the loss of infectivity in its natural host remains unknown.

## Data availability statement

The sequence datasets presented in this study can be found online. The names of the repository and accession number(s) can be found below: https://www.ncbi.nlm.nih.gov/ and OR348434. Other datasets presented are available upon request.

## Ethics statement

The animal study was approved by Iowa State University Institutional Animal Care and Use Committee and USDA-ARS Institutional Animal Care and Use Committee. The study was conducted in accordance with the local legislation and institutional requirements.

## Author contributions

PG: Methodology, Writing – original draft, Writing – review & editing. DC: Formal analysis, Methodology, Writing – review & editing. C-TX: Methodology, Writing – review & editing. QC: Methodology, Writing – review & editing. KL: Conceptualization, Funding acquisition, Methodology, Resources, Writing – review & editing. BB: Resources, Writing – review & editing. X-JM: Methodology, Writing – review & editing. PH: Methodology, Writing – review & editing. TO: Conceptualization, Methodology, Project administration, Supervision, Writing – original draft, Writing – review & editing.

## References

[1] Jung K Saif LJ Wang Q. Porcine epidemic diarrhea virus (PEDV): an update on etiology, transmission, pathogenesis, and prevention and control. Virus Res. (2020) 286:198045. doi: 10.1016/j.virusres.2020.198045, PMID: 32502552 PMC7266596

[2] Woo PCY de Groot RJ Haagmans B Lau SKP Neuman BW Perlman S. ICTV virus taxonomy profile: *Coronaviridae* 2023. J Gen Virol. (2023) 104. doi: 10.1099/jgv.0.001843, PMID: 37097842 PMC12135074

[3] Guo J Fang L Ye X Chen J Xu S Zhu X. Evolutionary and genotypic analyses of global porcine epidemic diarrhea virus strains. Transbound Emerg Dis. (2019) 66:111–8. doi: 10.1111/tbed.12991, PMID: 30102851 PMC7168555

[4] Kim SJ Nguyen VG Huynh TM Park YH Park BK Chung HC. Molecular characterization of porcine epidemic diarrhea virus and its new genetic classification based on the nucleocapsid gene. Viruses. (2020) 12:790. doi: 10.3390/v12080790, PMID: 32717934 PMC7472284

[5] Pensaert MB Martelli P. Porcine epidemic diarrhea: a retrospect from Europe and matters of debate. Virus Res. (2016) 226:1–6. doi: 10.1016/j.virusres.2016.05.030, PMID: 27317168 PMC7132433

[6] Debouck P Pensaert M. Experimental infection of pigs with a new porcine enteric coronavirus, CV 777. Am J Vet Res. (1980) 41:219–23. PMID: 6245603

[7] Wang D Fang L Xiao S. Porcine epidemic diarrhea in China. Virus Res. (2016) 226:7–13. doi: 10.1016/j.virusres.2016.05.026, PMID: 27261169 PMC7114554

[8] Hanke D Pohlmann A Sauter-Louis C Hoper D Stadler J Ritzmann M. Porcine epidemic diarrhea in Europe: in-detail analyses of disease dynamics and molecular epidemiology. Viruses. (2017) 9:177. doi: 10.3390/v9070177, PMID: 28684708 PMC5537669

[9] Chen Q Gauger PC Stafne MR Thomas JT Madson DM Huang H. Pathogenesis comparison between the United States porcine epidemic diarrhoea virus prototype and S-INDEL-variant strains in conventional neonatal piglets. J Gen Virol. (2016) 97:1107–21. doi: 10.1099/jgv.0.000419, PMID: 26841768

[10] Jung K Annamalai T Lu Z Saif LJ. Comparative pathogenesis of US porcine epidemic diarrhea virus (PEDV) strain PC21A in conventional 9 days-old nursing piglets vs. 26 days-old weaned pigs. Vet Microbiol. (2015) 178:31–40. doi: 10.1016/j.vetmic.2015.04.022, PMID: 25939885 PMC7117181

[11] Lin CM Annamalai T Liu X Gao X Lu Z El-Tholoth M. Experimental infection of a US spike-insertion deletion porcine epidemic diarrhea virus in conventional nursing piglets and cross-protection to the original US PEDV infection. Vet Res. (2015) 46:134. doi: 10.1186/s13567-015-0278-9, PMID: 26589292 PMC4654902

[12] Yamamoto R Soma J Nakanishi M Yamaguchi R Niinuma S. Isolation and experimental inoculation of an S INDEL strain of porcine epidemic diarrhea virus in Japan. Res Vet Sci. (2015) 103:103–6. doi: 10.1016/j.rvsc.2015.09.024, PMID: 26679803 PMC7173067

[13] Lazov CM Lohse L Belsham GJ Rasmussen TB Botner A. Experimental infection of pigs with recent European porcine epidemic diarrhea viruses. Viruses. (2022) 14:2751. doi: 10.3390/v14122751, PMID: 36560755 PMC9780976

[14] Gallien S Moro A Lediguerher G Catinot V Paboeuf F Bigault L. Limited shedding of an S-InDel strain of porcine epidemic diarrhea virus (PEDV) in semen and questions regarding the infectivity of the detected virus. Vet Microbiol. (2019) 228:20–5. doi: 10.1016/j.vetmic.2018.09.025, PMID: 30593368 PMC7117288

[15] Lohse L Krog JS Strandbygaard B Rasmussen TB Kjaer J Belsham GJ. Experimental infection of young pigs with an early European strain of porcine epidemic diarrhoea virus and a recent US strain. Transbound Emerg Dis. (2017) 64:1380–6. doi: 10.1111/tbed.12509, PMID: 27161288 PMC7169680

[16] Egberink HF Ederveen J Callebaut P Horzinek MC. Characterization of the structural proteins of porcine epizootic diarrhea virus, strain CV777. Am J Vet Res. (1988) 49:1320–4. PMID: 3140695

[17] Chen Q Li G Stasko J Thomas JT Stensland WR Pillatzki AE. Isolation and characterization of porcine epidemic diarrhea viruses associated with the 2013 disease outbreak among swine in the United States. J Clin Microbiol. (2014) 52:234–43. doi: 10.1128/JCM.02820-13, PMID: 24197882 PMC3911415

[18] Thomas JT Chen Q Gauger PC Gimenez-Lirola LG Sinha A Harmon KM. Effect of porcine epidemic diarrhea virus infectious doses on infection outcomes in naive conventional neonatal and weaned pigs. PLoS One. (2015) 10:e0139266. doi: 10.1371/journal.pone.0139266, PMID: 26441071 PMC4594914

[19] Gerber PF Xiao CT Lager K Crawford K Kulshreshtha V Cao D. Increased frequency of porcine epidemic diarrhea virus shedding and lesions in suckling pigs compared to nursery pigs and protective immunity in nursery pigs after homologous re-challenge. Vet Res. (2016) 47:118. doi: 10.1186/s13567-016-0402-5, PMID: 27871312 PMC5118895

[20] Gerber PF Gong Q Huang YW Wang C Holtkamp D Opriessnig T. Detection of antibodies against porcine epidemic diarrhea virus in serum and colostrum by indirect ELISA. Vet J. (2014) 202:33–6. doi: 10.1016/j.tvjl.2014.07.018, PMID: 25135339 PMC7110509

[21] Gerber PF Opriessnig T. Detection of immunoglobulin (Ig) a antibodies against porcine epidemic diarrhea virus (PEDV) in fecal and serum samples. MethodsX. (2015) 2:368–73. doi: 10.1016/j.mex.2015.10.001, PMID: 26587386 PMC4625113

[22] Stevenson GW Hoang H Schwartz KJ Burrough ER Sun D Madson D. Emergence of porcine epidemic diarrhea virus in the United States: clinical signs, lesions, and viral genomic sequences. J Vet Diagn Investig. (2013) 25:649–54. doi: 10.1177/1040638713501675, PMID: 23963154

[23] Kim O Chae C Kweon C-H. Monoclonal antibody-based immunohistochemical detection of porcine epidemic diarrhea virus antigen in formalin-fixed, paraffin-embedded intestinal tissues. J Vet Diagn Investig. (1999) 11:458–62. doi: 10.1177/104063879901100512, PMID: 12968761

[24] St John SE Anson BJ Mesecar AD. X-ray structure and inhibition of 3C-like protease from porcine epidemic diarrhea virus. Sci Rep. (2016) 6:25961. doi: 10.1038/srep25961 27173881 PMC4865815

[25] Ye G Wang X Tong X Shi Y Fu ZF Peng G. Structural basis for inhibiting porcine epidemic diarrhea virus replication with the 3C-like protease inhibitor GC376. Viruses. (2020) 12:240. doi: 10.3390/v12020240, PMID: 32098094 PMC7077318

[26] Bridgen A Duarte M Tobler K Laude H Ackermann M. Sequence determination of the nucleocapsid protein gene of the porcine epidemic diarrhea virus confirms that this virus is a coronavirus related to human coronavirus 229E and porcine transmissible gastroenteritis virus. J Gen Virol. (1993) 74:1795–804. doi: 10.1099/0022-1317-74-9-1795, PMID: 8397280

[27] de Arriba ML Carvajal A Pozo J Rubio P. Mucosal and systemic isotype-specific antibody responses and protection in conventional pigs exposed to virulent or attenuated porcine epidemic diarrhoea virus. Vet Immunol Immunopathol. (2002) 85:85–97. doi: 10.1016/s0165-2427(01)00417-2, PMID: 11867170

[28] Coussement W Ducatelle R Debouck P Hoorens J. Pathology of experimental CV777 coronavirus enteritis in piglets. I. Histological and histochemical study. Vet Pathol. (1982) 19:46–56. doi: 10.1177/030098588201900108, PMID: 6280359

[29] Kweon CH Kwon BJ Lee JG Kwon GO Kang YB. Derivation of attenuated porcine epidemic diarrhea virus (PEDV) as vaccine candidate. Vaccine. (1999) 17:2546–53. doi: 10.1016/s0264-410x(99)00059-6, PMID: 10418901 PMC7125954

[30] Beall A Yount B Lin CM Hou Y Wang Q Saif L. Characterization of a pathogenic full-length cDNA clone and transmission model for porcine epidemic diarrhea virus strain PC22A. mBio. (2016) 7:e01451–15. doi: 10.1128/mBio.01451-15, PMID: 26733065 PMC4724997

[31] Li W van Kuppeveld FJM He Q Rottier PJM Bosch BJ. Cellular entry of the porcine epidemic diarrhea virus. Virus Res. (2016) 226:117–27. doi: 10.1016/j.virusres.2016.05.031, PMID: 27317167 PMC7114534

[32] Cox E Pensaert MB Callebaut P van Deun K. Intestinal replication of a porcine respiratory coronavirus closely related antigenically to the enteric transmissible gastroenteritis virus. Vet Microbiol. (1990) 23:237–43. doi: 10.1016/0378-1135(90)90154-N 2169676 PMC7117313

[33] Kipar A Meli ML. Feline infectious peritonitis: still an enigma? Vet Pathol. (2014) 51:505–26. doi: 10.1177/0300985814522077, PMID: 24569616

