# Supplementary material for: Failure to experimentally infect 10 days-old piglets with a cell culture-propagated infectious stock of a classical genotype 1a porcine epidemic diarrhea virus
Source: Front Vet Sci. 2023 Nov 16;10:1279162. doi: 10.3389/fvets.2023.1279162 (PMC10693406; doi:10.3389/fvets.2023.1279162)
Supplement: Supplementary file 1 [file Data_Sheet_1.pdf]

Table S1: Primers for sequencing of CV777 genomic DNA

Primers	Sequences	Primers	Sequences
PEDV618R21	5' CAACTGGCACGATGTTACCAC 3'	PEDV13973F20	5' TTTGCCAAGCTCGCGTCGTG 3'
PEDV1248F21	5' TYAAGGTGCAGTCCAAAGACG 3'	PEDV14797F21	5' TTCGTCGTTGAGTATTATGGT 3'
PEDV1373R21	5' AGAAAACTTGCCACTAAGGAT 3'	PEDV14999R21	5' GCGAGCAAAATTCATGAGGAC 3'
PEDV1899F21	5' AAAAYGCCAATGTGAATCTCG 3'	PEDV15382F21	5' CTTTGTGTTGTTTGTGGCTCT 3'
PEDV2557F21	5' AGCACGCAAATTTAAACGACT 3'	PEDV16003F21	5' ACATATAAAACTACCGCCACA 3'
PEDV2650R22	5' GGTGGCATAATACTTGAAGCTA 3'	PEDV17327R21	5' AACGGAAACCCATAAACGAGA 3'
PEDV3101F21	5' YGTATCAAGGTTACAGGTGGT 3'	PEDV17502F21	5' CGGTGTTGATTTTGTTGTCAG 3'
PEDV3676F21	5' GTCACAAAAGGCTATYTTGGG 3'	PEDV17909R21	5' GGTTAAGGCTAAGTGATCCCT 3'
PEDV4866R23	5' GCYRGTCACATYGTTAAGACACT 3'	PEDV18155F20	5' TTGGCAATCCTAAGGGCATT 3'
PEDV5260F21	5' ACAACAAATTGGACCTTGTCT 3'	PEDV18531F21	5' TCTTAGGGCTAGTAACTGCAT 3'
PEDV5414R21	5' TTCRCTAGGAAAGGYAAACCC 3'	PEDV19275F21	5' CCAAGGTAGAACAACCGCTGA 3'
PEDV6571R20	5' GGTGTTGCCATACWACCTGT 3'	PEDV19386R21	5' TCACCATACACAACGTGCTCA 3'
PEDV6936F21	5' TCTACGTACATGCCAATGGTG 3'	PEDV19717F21	5' TATCCRCAACTTCARGCCAGT 3'
PEDV7089R22	5' TCAATAAGAATAGTGGCAGGAC 3'	PEDV20333F21	5' CAGAGGCATTYTTAATTGGTG 3'
PEDV7500F21	5' TTAATGCTGCTGTTGCTGAGG 3'	PEDV20900F20	5' TGAGATTGGCATTTCGCAAG 3'
PEDV8636R22	5' AAAGTATTTGACCAGACAACAC 3'	PEDV22026R21	5' ACACTTGAGTTGGCTAACAGG 3'
PEDV9005F21	5' GGYGACAAGTTCGTAGGCTCT 3'	PEDV22544F23	5' GGACGTTTCTTTTATGACTCTGG 3'
PEDV9179R22	5' CCAAATGGGCAAAACAAGCAAG 3'	PEDV22668R21	5' CTTAAAGGCTAACAACTGTCC 3'
PEDV9566F21	5' GTCCACACGCCTAAGTACACC 3'	PEDV23324F21	5' CYTGCTTTTTAATAAAGTGGT 3'
PEDV10111F21	5' AATTCTTGGCYATACCTCGTT 3'	PEDV23603R19	5' CAATTGCTGGTTCCGCTGT 3'
PEDV10727F21	5' GCTGTTTATATGGCCTTGAGA 3'	PEDV24394R21	5' TTCTATTGGGCAGAGAAGCTA 3'
PEDV10826R22	5' AGTAGAGAATACCGTAGAAGCA 3'	PEDV24778F21	5' ACGTGCAGTGATGTTTCTTGG 3'
PEDV11332F21	5' ACGCCAACAGTATGAAGATGC 3'	PEDV26078R22	5' CGCCAGTAGCAACCTTATAGCC 3'
PEDV11875F21	5' TATTCCYGGTAAGCTGAAGCA 3'	PEDV26653R21	5' AATTGGCTTTTCAGACGCCTT 3'
PEDV13012F22	5' ATTAAGGTAGGCGCTTGTGAGG 3'	PEDV27225R21	5' TCGACAAATTCCGCATCTCCA 3'
PEDV13107R21	5' GCACGTGAAACAATGGTACCT 3'	PEDV27467F22	5' AGGAAAAGAAGAACAAGCGTGA 3'
PEDV13631F21	5' GTATAGTTTGGAAYAATGACC 3'	PEDV27830R22	5' GAAAAGGTACTGCGTTCCCCTC 3'

Supplementary Table 2. List of primers for RT-PCR of CV777 genome.

Primer Set	Primers	Sequence	Size
A	PEDV1F30	5' ACTTAAAAAGATTTTCTATCTACGGATAGT 3'	2052
	PEDV2053R22	5' CTTGAGCTCACAAGCAGACTTA 3'	
B	PEDV1988F22	5' TTCGAGAGTGACGGGTTTTACA 3'	2208
	PEDV4196R24	5' AAGGTCACTAAAATCTCCTTGGTA 3'	
C	PEDV4057F25	5' TCGTCATCAGATAAAGTATGACACA 3'	1863
	PEDV5920R24	5' CCGTTACCATTATCTAGGAATGTT 3'	
D	PEDV5842F25	5' AGGTACTACAATTGTTGTCAATGTT 3'	2362
	PEDV8204R23	5' AAGCACAAACCAGATGTACCAAA 3'	
E	PEDV8074F23	5' TTTCACCCCCGTCAACAATCCTA 3'	2607
	PEDV10681R23	5' CGAACCAGTTGCCAGTCACACTA 3'	
F	PEDV10502F23	5' GCACAAGGTCTTGTTAACATCTT 3'	2112
	PEDV12614R23	5' TGCACTAGAGCCCCGTACTCGTT 3'	
G	PEDV12538F23	5' GGTTTGTGGTTGTTGGCTGGCTA 3'	2060
	PEDV14598R23	5' AGGTAGTACCACCTGGCTTCAAA 3'	
H	PEDV14442F23	5' CGACAGAGCACTGCCCAATATGA 3'	2218
	PEDV16660R23	5' ACATCAGGCTTAAGGGCACACAT 3'	
I	PEDV16538F23	5' ATCAGCGCATCAGCTATAGGCAT 3'	2486
	PEDV19024R23	5' ACGTCACCCTCAAAGTCGGTATA 3'	
J	PEDV18922F23	5' ACGATCCTACGTAACTTGGGTGT 3'	2695
	PEDV21617R23	5' GAAGTACAATTGAGCCTTCAGCA 3'	
K	PEDV21482F25	5' GGCCATTCCTAAGATTTATGGACTA 3'	2486
	PEDV23968R23	5' CTCATTAACCTTTTGCTGTGCTA 3'	
L	PEDV23845F23	5' GACTGGACATTCTTTCAGCCGAT 3'	1928
	PEDV25773R25	5' ACAAGAGGCCAAAGTATCCATAGAA 3'	
M	PEDV25496F25	5' GCTTCACTTGTCACCGGTTGTGTAA 3'	2451
	PEDV27947R27	5' GTGTATCCATATCAACACCGTCAGGTC 3'	
